# ITSN2L Interacts with and Negatively Regulates RABEP1

**DOI:** 10.3390/ijms161226091

**Published:** 2015-11-27

**Authors:** Xiaoxu Yang, Feng Yan, Zhicheng He, Shan Liu, Yeqing Cheng, Ke Wei, Shiquan Gan, Jing Yuan, Shang Wang, Ye Xiao, Kaiqun Ren, Ning Liu, Xiang Hu, Xiaofeng Ding, Xingwang Hu, Shuanglin Xiang

**Affiliations:** 1 Key Laboratory of Protein Chemistry and Developmental Biology of State Ministry of Education, College of Life Sciences, Hunan Normal University, Changsha 410081, China; yangxiaoxu@hunnu.edu.cn (X.Y.); yanfeng1944@aliyun.com (F.Y.); huzech@gmail.com (Z.H.); ls198804180303@sina.com (S.L.); chengfily@gmail.com (Y.C.); weike8629@163.com (K.W.); qingfusanduan@163.com (S.G.); yuanjing27@126.com (J.Y.); wangshang887@gmail.com (S.W.); xy5488@163.com (Y.X.); kaiqunren@126.com (K.R.); liuning0731@126.com (N.L.); huxiang@hunnu.edu.cn (X.H.); fengxiaoding@hotmail.com (X.D.); 2 Department of Dermatology, Xiangya Hospital, Central South University, Changsha 410081, China

**Keywords:** ITSN2L, RABEP1, endocytosis, endosome, interactions

## Abstract

Intersectin-2Long (ITSN2L) is a multi-domain protein participating in endocytosis and exocytosis. In this study, RABEP1 was identified as a novel ITSN2L interacting protein using a yeast two-hybrid screen from a human brain cDNA library and this interaction, specifically involving the ITSN2L CC domain and RABEP1 CC3 regions, was further confirmed by *in vitro* GST (glutathione-*S*-transferase) pull-down and *in vivo* co-immunoprecipitation assays. Corroboratively, we observed that these two proteins co-localize in the cytoplasm of mammalian cells. Furthermore, over-expression of ITSN2L promotes RABEP1 degradation and represses RABEP1-enhanced endosome aggregation, indicating that ITSN2L acts as a negative regulator of RABEP1. Finally, we showed that ITSN2L and RABEP1 play opposite roles in regulating endocytosis. Taken together, our results indicate that ITSN2L interacts with RABEP1 and stimulates its degradation in regulation of endocytosis.

## 1. Introduction

**Figure 1:**
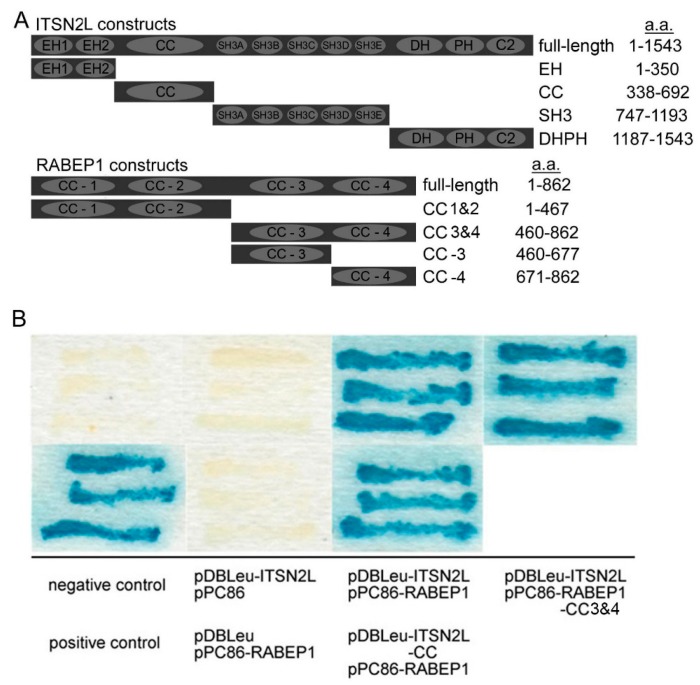

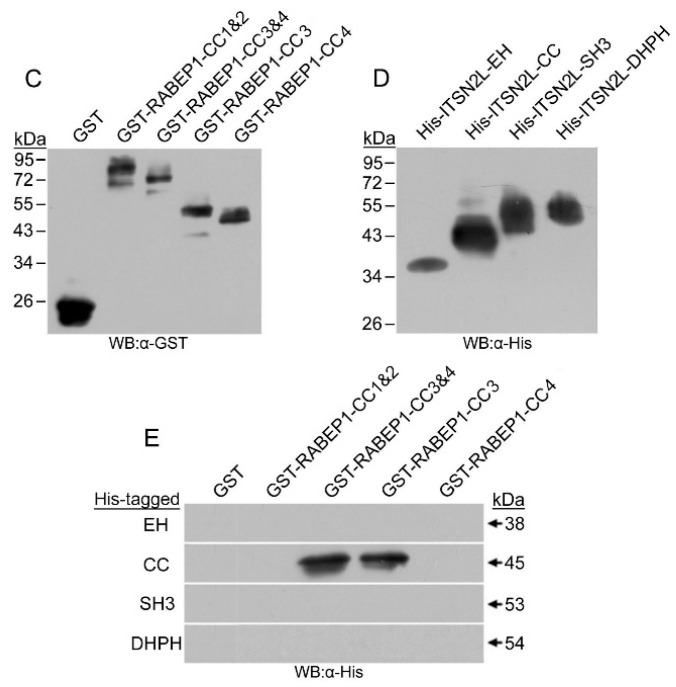
Identification of Intersectin-2Long-coiled-coil (ITSN2L-CC) domain-interacting partners by Y2H screen and verification of interaction between ITSN2L and rabaptin-5 (RABEP1) *in vitro*. (**A**) Schematic representation of the domain structure of ITSN2L and RABEP1 used in this study; (**B**) The colonies were grown on SC-Leu-Trp-His+30 mM AT plates after transformation with pDBLeu-ITSN2L-CC and pPC86-human brain cDNA library and then duplicated onto a nylon membrane for the Beta-Gal assay. Positive clones were confirmed by retransformation and sequences were further analyzed; (**C**) Purified GST-RABEP1 fusion detected by anti-GST antibody; (**D**) Purified His-ITSN2L fusion detected by anti-His antibody; (**E**) GST pull-down assays analyzing interaction between these subdomains of ITSN2L and RABEP1. Proteins pulled down were detected by anti-His antibody.

Human intersectins (including ITSN1 and ITSN2) are conserved proteins playing essential roles in cell growth and development. These multi-domain scaffold proteins are comprised of two NH_2_-terminal Eps15 homology (EH) domains, a coiled-coil (CC) region, five Src homology 3 (SH3) domains, a Dbl homology (DH) domain, a pleckstrin homology (PH) domain and a C2 domain (Figure 1A). To date, many proteins have been found to interact with ITSNs. For example, the EH domain has been reported to interact with epsin [1]; the CC domain bind Eps15 and SNAP-25 [2,3]; the SH3 domain can interact with N-WASP, Sos, Cbl, dynamin and synaptojanin [1,3,4,5,6]; the PH domain can interact with lipid and cell membrane [7]. ITSN1 was reported to participate in Clathrin-mediated endocytosis [2], synaptic vesicle recycling [8] and regulate MAPK (mitogen-activated protein kinase) signaling through inhibition of Ras activation by interacting with Sos [9]. More detailed reviews can be found elsewhere [10]. Both ITSN1 and 2 possess short and long isoforms. ITSN2L, the larger (~190 kDa) isoform of ITSN2, was first described by Hussain *et al.* in 1999 [11] and was reported to be involved in clathrin [12] and caveola [13] mediated endocytosis. Both ITSN2 short and long forms co-localize with Eps15 and show a fine punctate pattern similar to other components of clathrin-coated vesicles [14]. ITSN2, as well as ITSN1, regulates actin polymerization via interaction with Cdc42 and N-wasp [13,15]. These findings demonstrate that ITSN2 links endocytosis machinery to actin cytoskeletal rearrangement. Unlike ITSN1 which is predominantly expressed in human brain [11], ITSN2 is widely expressed, although the patterns of long and short isoforms are different [16]. Compared to its short isoform, the ITSN2L possess extra DH and PH domains [2]. These extra domains have been reported to serve as guanine nucleotide exchange factor (GEF) for Rho GTPase such as Cdc42 [13]. GEF activity of DH domain has been attributed to Cdc42 activation and the subsequent actin relocation and changes in cell morphology [13]. Others have reported that ITSN2L recruits Cdc42 and WASP to endocytic vesicles in T cells during T cell receptor (TCR) endocytosis, and this also requires GEF activity of DH domain [17]. More recently, ITSN2 was reported to localize to centrosomes, recruiting and activating Cdc42 through the DH domain. It regulates spindle orientation during mitosis [18].

To understand the function of ITSN2, we employed the yeast two-hybrid (Y2H) screen using coiled-coil domain of ITSN2L as bait to identify potential interacting proteins. Novel putative interacting proteins identified (Table S1) include EPS15L1, EPS8 and RABEP1 (rabaptin-5). We are interested in RABEP1 because it is an essential and rate-limiting component of the endosome fusion that regulates early endosomal transport through interaction with small Ras-related GTPases, Rab4 and Rab5 [19,20]. The Rab GTPasesare essential regulators of intracellular transport [21] which are bound with their Rab GTP-dissociation inhibitor (GDI) before being delivered to the right destination [22]. When recruited to clathrin coated pits, Rab5 was activated by a complex comprised of GEFs, Rabex5 and RABPE1 [23] engaging endosome fusion. After membrane fusion, GTPase activating factors (GAPs) transiently interact with Rab and stimulate GTP hydrolysis [24] resulting in Rab5 inactivation [25] and release to the cytosol, which allow GDI recognition for recycling [26]. Rab5 regulates fusion between homotypic early endosomes [27]. It recruits RABEP1 to early endosomes in a GTP-dependent manner, while immunodepletion of RABEP1 strongly inhibits Rab5 dependent early endosome fusion [19]. Thus, RABEP1 functions as a vital regulator and molecular switch for Rab5 function and, therefore, controls vesicular cargo delivery. However, less is known about the recruiting and regulating mechanisms of this protein that contains four coiled-coil domains. Our finding of the interaction between ITSN2L and RABEP1 may bring new insights into endocytosis machinery and endosome trafficking.

## 2. Results

### 2.1. ITSN2L Interacts with RABEP1 Both in Vitro and Vivo

In order to identify ITSN2L interacting proteins, yeast strain MaV203 was sequentially transfected with pDBLeu-ITSN2L-CC and human brain cDNA library constructed with pPC86. Several ITSN2L-CC-interacting proteins including RABEP1 were identified (Table S1), RABEP1 was chosen for further study, because ITSN2L has been shown to participate in clathrin coated endocytosis budding, fission [12] and endosome transporting [13,28] and examining its interaction with RABEP1 may gain insights into its function during later stages of endocytosis-endosome fusion [19].

To confirm the interaction between ITSN2L and RABEP1, MaV203 yeast strain was transfected with combinations of pDBLeu-ITSN2L along with pPC86-RABEP1, pDBLeu-ITSN2L-CC with pPC86-RABEP1, and pDBLeu-ITSN2L with pPC86-RABEP1-CC3&4. The Beta-Gal assay shown in Figure 1B, blue colonies were observed only in cells containing pDBLeu-ITSN2L and pPC86-RABEP1, or pDBLeu-ITSN2L-CC and pPC86-RABEP1, or pDBLeu-ITSN2L and pPC86-RABEP1-CC3&4, or in positive control containing pPC97-CYH2s and pPC86-dE2F (Figure 1B).

Physical interactions between ITSN2L and RABEP1 subdomains were then examined *in vitro* by GST pull-down assays. Truncated RABEP1 GST fusion and ITSN2L His fusion proteins (Figure 1A) were purified (Figure 1C). As shown in Figure 1D, the His-ITSN2L-CC fusion protein bound to the GST-RABEP1-CC3&4 and GST-RABEP1-CC3 fusion protein. In contrast, His-ITSN2L-EH/SH3/DHPH fusions did not show binding to GST-RABEP1-CC1&2 fusions. These results indicated that the interaction between these two proteins is introduced by the ITSN2L CC domain and RABEP1 CC3.

**Figure 2:**
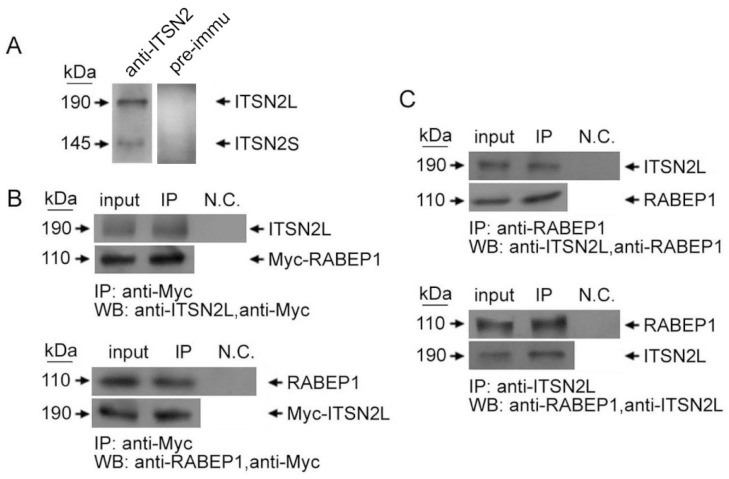
Co-immunoprecipitation of ITSN2L and RABEP1. (**A**) Rabbit ITSN2L recognize both human ITSN2 long and short isoforms; (**B**) HeLa cells were transfected with Myc-RABEP1 or Myc-ITSN2L, respectively, and cell lysates were subjected to immunoprecipitation using rabbit polyclonal anti-Myc or rabbit preimmune IgG. The IP materials were analyzed with anti-ITSN2L (**upper** panel) or anti-RABEP1 (**lower** panel), respectively; (**C**) Lysates from HeLa cells were subjected to IP using mouse monoclonal anti-RABEP1 or rabbit pre-immune IgG and immunoblotted with rabbit polyclonal anti-ITSN2L (**uppe**r panel), or IP using rabbit polyclonal anti-ITSN2L or rabbit preimmune IgG and immunoblotted with mouse monoclonal anti-RABEP1 (**lower** panel).

ITSN2L and RABEP1 interaction was then further tested *in vivo* by immunoprecipitation (IP). HeLa cells were transiently transfected with pCMV-Myc-ITSN2L or pCMV-Myc-RABEP1. Lysates were immunoprecipitated by rabbit polyclonal antibodies against Myc-tag, and then detected by rabbit polyclonal antibodies against ITSN2L or mouse monoclonal antibodies against RABEP1, respectively. ITSN2L can be precipitated by anti-Myc antibody, but rabbit normal IgG did not detect any band (Figure 2B, upper panel). Likewise, RABEP1 can be immunoprecipitated with Myc-ITSN2L, whereas mouse normal IgG did not recognize any band (Figure 2B, lower panel). Meanwhile, endogenous RABEP1 and ITSN2L interaction was also tested. Endogenous RABEP1 could be precipitated together with ITSN2L but not by negative control rabbit IgG and the same result obtained when the experiment was performed conversely (Figure 2C). Therefore, these data ascertained that RABEP1 and ITSN2L interact *in vivo*. Since CC domain of ITSN2L is a multi-protein interacting domain, another Myc-tagged CC domain binding protein EPS8 [28] was overexpressed in HeLa cell. Quantitative immunoprecipitation of ITSN2L and follows RABEP1 detection was carried out; data showed that excessive EPS8 can inhibit the interaction between CC-ITSN2L and RABEP1 in a competitive manner.

### 2.2. ITSN2L Co-Localizes with RABEP1

**Figure 3:**
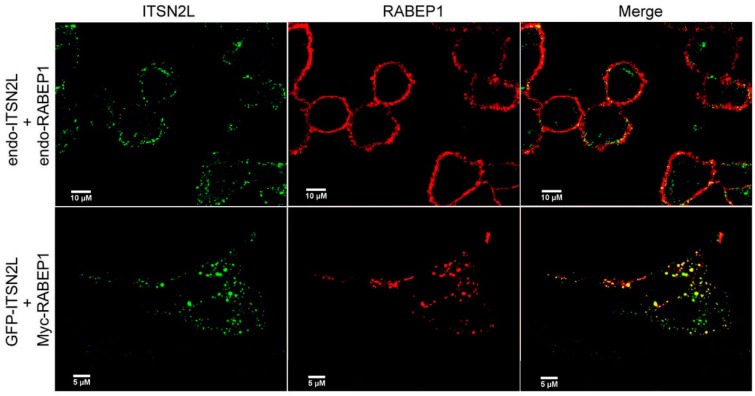
Co-localization of ITSN2L and RABEP1 in HeLa cells. **Uppe**r panels show localization of endogenous ITSN2L and RABEP1, ITSN2L was detected by rabbit polyclonal anti-ITSN2L antibody and Alexa-488 goat anti-rabbit secondary antibody, and RABEP1 by mouse monoclonal anti-RABEP1 antibody and Alexa-594 goat anti-mouse secondary antibody; **Lower** panels show localization of GFP-ITSN2L and Myc-RABEP1, detected with mouse monoclonal anti-Myc antibody and Alexa-594 conjugated goat anti-mouse secondary antibody.

Based on the ITSN2L and RABEP1 interaction observed, we investigated the subcellular distribution of these two proteins *in vivo*. HeLa cells were cultured on glass coverslips in 12 well plates and stained by anti-ITSN2L and anti-RABEP1 antibodies. Both ITSN2L and RABEP1 are localized on cell membrane (Figure 3, upper panels). As for the exogenous protein, HeLa cells were transfected with GFP-ITSN2L along with Myc-RABEP1, subsequently stained with anti-Myc antibodies. We found that Myc-RABEP1 distribution shows a complete overlap with GFP-ITSN2L in a scattered cluster pattern both on membrane and in cytoplasm (Figure 3, lower panels). In addition, cytoplasmic ITSN2L shows co-localization with EEA1, an early endosome marker (Figure S1). Their distribution pattern was similar to Myc-RABEP1. This may indicate a possible function of ITSN-RABEP1 interaction related to early endosomes.

### 2.3. ITSN2L Acts as a Negative Regulator of RABEP1

**Figure 4:**
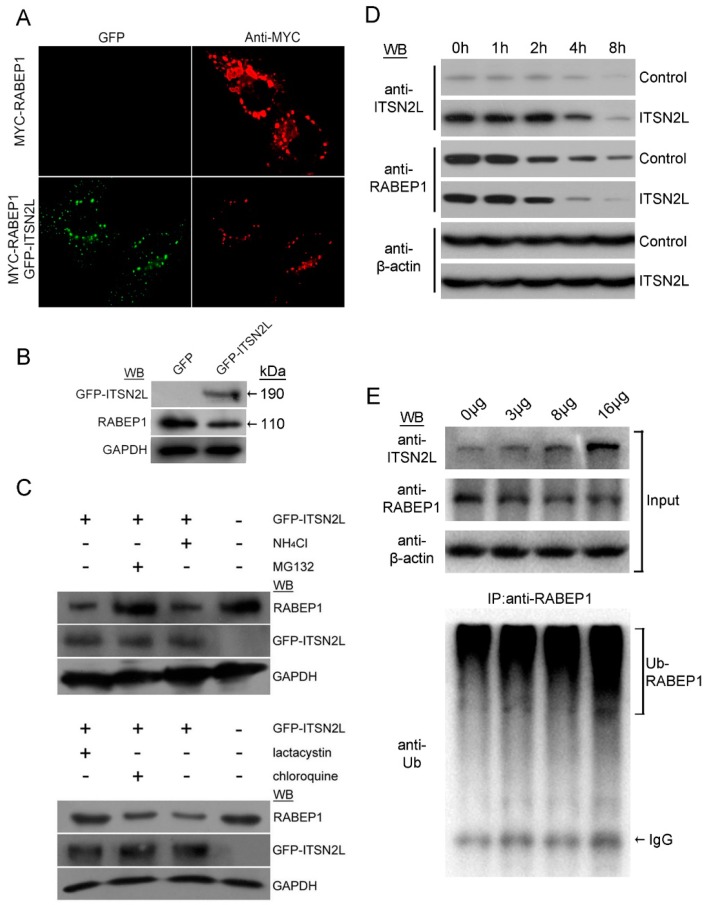
ITSN2L inhibits the accumulation of vesicles induced by Myc-RABEP1 and promotes RABEP1 degradation. (**A**) HeLa cells transfected with Myc-RABEP1show the accumulation of large vesicles, while Myc-RABEP1co-transfected with GFP-ITSN2L revealed reduced large vesicles. The images were taken at a magnification of 600; (**B**) Expression of endogenous RABEP1 was reduced when cells over expressing ITSN2L HeLa cells were transfected with GFP or GFP-ITSN2L, cell lysates were detected with anti-GFP, anti-RABEP1 and anti-GAPDH antibodies; (**C**) The induced degradation can be rescued by proteasome inhibitors. HeLa cells pre-treated with NH_4_Cl (10 mM), MG132 (20 μM), lactacystin (20 μM), and chloroquine (100 μM) were then transfected with GFP-ITSN2L with inhibitors present throughout; (**D**) Half-life of RABEP1 protein after ITSN2L Overexpression. HeLa cells transfected with GFP-ITSN2L or GFP were incubated with cycloheximide and time points were taken. Cells of each sample were then lysed and detected by the indicated antibodies; (**E**) Ubiquitination of RABEP1 upon ITSN2L overexpressed. HeLa cells transfected with indicated amount of GFP-ITSN2L were lysed and the same amount of total protein used for immunoprecipitation of RABEP1 and detected by the indicated antibodies.

Previous reports revealed that RABEP1 overexpression can alter endosome morphology [19]. Consistently, we observed that HeLa cells transfected with Myc-RABEP1 could induce huge endosomes. Surprisingly, when Myc-RABEP1 co-expressed with GFP-ITSN2L, endosomes mostly split into small vesicles in cytoplasm and abort assembling together (Figure 4A).These results indicate that ITSN2L may effect on RABEP1 mediated vesicles trafficking. In order to investigate the relationship between ITSN2L and endogenous RABEP1, GFP-ITSN2L was overexpressed in HeLa cells, and detected by RABEP1 antibodies. As shown in Figure 4B, expression of RABEP1 was suppressed by GFP-ITSN2L overexpression, suggesting that ITSN2L may act as a negative regulator of RABEP1.

Previous studies have demonstrated that ITSN could function as scaffold for E3 ubiquitin ligase [6]. However, it is not clear which pathway was involved in the reduction of RABEP1 induced by ITSN2L. To investigate this, GFP-ITSN2L overexpressing cells were pre-treated with lysosome inhibitor NH_4_Cl, chloroquine or proteasome inhibitor MG132, lactacystin to examine the inhibition effects on RABEP1. These inhibition assays revealed that suppression of RABEP1 by ITSN2L could be reversed by proteasome inhibitor MG132 and lactacystin, but not lysosome inhibitor NH_4_Cl and chloroquine (Figure 4C). Meanwhile, the protein half-life of RABEP1 under GFP-ITSN2L overexpressing was measured. GFP or GFP-ITSN2L transfected HeLa cells were treated with 100 μg/mL of cycloheximide 24 h post transfection for 1, 2, 4, or 8 h. Harvested samples were detected by ITSN2L and RABEP1 antibodies. Data showed RABEP1 degradation was significantly accelerated (Figure 4D). Furthermore, the ubiquitination status was also tested. HeLa cells in 10 cm dishes were transfected with GFP or 3, 8, 16 μg of GFP-ITSN2L plus 3 µg of Myc-Ub plasmid. Control GFP vector was used as compensation for each group (up to 19 µg total per transfection). 20 μM of MG123 were added 48 h post transfection for 5 h. Immunoprecipitations of RABEP1 were then performed by using equal amount of protein extracted from each sample. Precipitates were then detected by anti Ub and RABEP1 antibodies (Figure 4E).The result indicated an increase of RABEP1 ubiquitination after ITSN2L overexpressing in a dose-dependent manner. In summary, our data demonstrated that ITSN2L promote RABEP1 ubiquitination and degradation through proteasome pathway.

**Figure 5:**
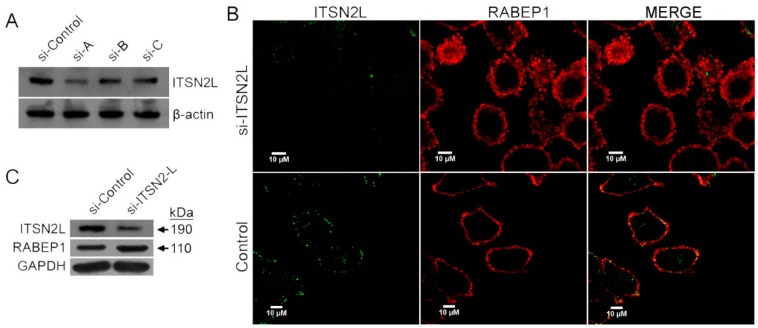
ITSN2L knockdown induce up-regulation of RABEP1. (**A**) HeLa cells transfected with siRNA-A, B, C and control respectively. Cell lysates were detected by western blotting with anti-ITSN2L and anti-β-actin antibody; (**B**) HeLa cells transfected with siRNA-A and control, subsequently stained with anti-ITSN2L and anti-RABEP1 antibody. Photos of si-RNA and control samples were taken under the same conditions of exposure; (**C**) HeLa cells transfected with siRNA-A and control. Cell lysates were detected by anti-ITSN2L, anti-RABEP1 and anti-GAPDH antibodies.

To further analyze the effect of ITSN2L on RABEP1 expression, RNAi was applied. Three siRNA sequences targeting different locations of conserved coiled-coil domain within ITSN2L were synthesized and transfected into HeLa cells respectively. Quantitative western blotting (Figure 5A) show that expression of ITSN2L was reduced by 25% (si-B, C) to 60% (si-A) compared to the control. Sequence A, which has most potent silencing effect, were chosen for further knockdown experiments. Cells transfected with ITSN2L siRNA-A was then stained with anti-ITSN2L and -RABEP1 antibodies for immunofluorescent analysis. The result showed that depletion of ITSN2L induces accretion of RABEP1 in the cytoplasm (Figure 5B). Consistently, this accumulation was also detected by western blotting (Figure 5C).

### 2.4. ITSN2L and RABEP1 Play Opposite Roles in Regulating Endocytosis

**Figure 6:**
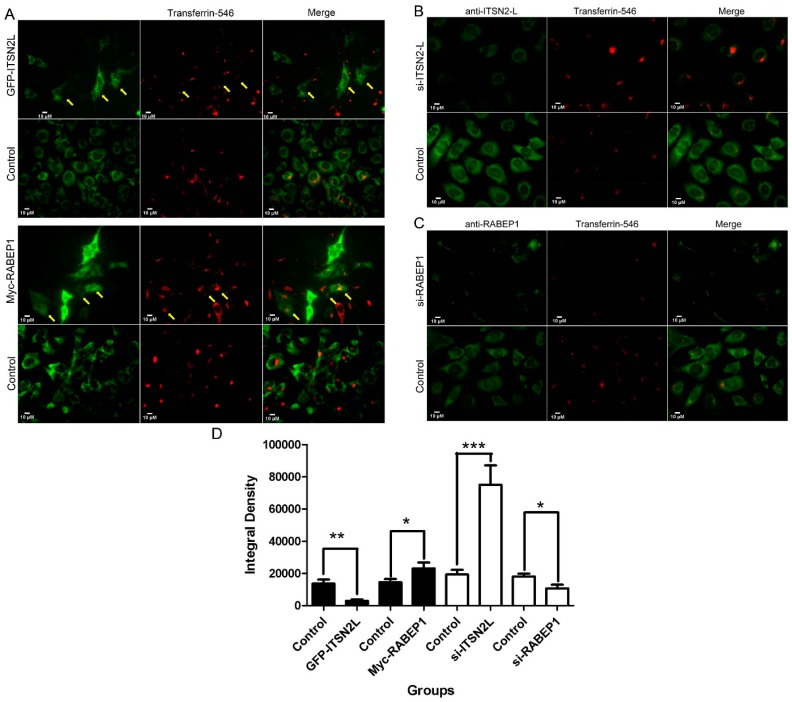
ITSN2L and RABEP1 play opposite roles in Transferrin uptake. (**A**) HeLa cells were transfected with GFP-ITSN2L or Myc-RABEP1. Overexpression of GFP-ITSN2L inhibited Tf uptake, while Myc-RABEP1 enhanced Tf uptake. Representative cells were marked by yellow arrows; (**B**) Knock down ITSN2L using si-RNA enhances the uptake of Tf; (**C**) Reduced RABEP1 expression caused a strong inhibition of Tf internalization. Pictures of si-RNA and control were acquired under the same exposure conditions; (**D**) Tf uptake signals of each group were measured with integral densities of red channel by imageJ and statistical analysis were carried out by *t*-test (*** *p* < 0.001, ** *p* < 0.01, * *p* < 0.05).

To evaluate the function of RABEP1 and ITSN2L in endocytosis, we performed Transferrin (Tf)-uptake assays. Cells expressing Myc-RABEP1 or GFP-ITSN2L were incubated with 15 μg/mL Alexa-546 Tf at 37 °C for 15 min. After washing and 4% paraformaldehyde fixing, cells were labeled by anti-Myc antibody. Gene expression and Tf uptake signals were then observed under fluorescent microscopy. As shown in Figure 6A and Figure S2, Tf uptake was attenuated in GFP-ITSN2L-transfectd cells as compared with empty vector transfected control (upper panels), while cells overexpressing Myc-RABEP1 showed enhanced Tf uptake (lower panels).

To further verify these findings, we performed Tf uptake assays after ITSN2L or RABEP1 knock down. Cells transfected with negative control, ITSN2L or RABEP1 si-RNA were then subjected to Alexa-546 Tf uptake and stained by anti-ITSN2L or -RABEP1 antibodies (Figure 6B,C). The results clearly showed that ITSN2L knockdown could enhance Tf endocytosis, while knockdown of RABEP1 caused strong inhibition of Tf internalization. Tf uptake signals of overexpressed or silenced cells (green cells) were cycled out and red signal of Tf was measured by imageJ as previous reported [29]. The result is consistent with the previous findings (Figure 6D).

Collectively, the overexpression and knockdown experiments suggested that ITSN2L and RABEP1 play opposite roles in regulating endocytosis of Tf. Moreover, ITSN2L might regulate endocytosis directly or indirectly through down-regulation of RABEP1.

## 3. Discussion

ITSN is a highly conserved protein. It is essential for cell growth and development. Despite many studies were carried out to examine the function of this multi-domain protein, such as signal transduction [30], receptor degradation [6], vesicle fission [31] and cell survival [32], its molecular interactions with other proteins are not well characterized. The CC domain of ITSN is shown to interact with many other proteins [10]. Thus, we decided to identify the interacting proteins of this highly conserved domain by using it as bait in Y2H screen. For the first time, we identified RABEP1 as an ITSN2L-interacting protein by screening a human brain cDNA library. The interaction between these two proteins was confirmed by GST pull-down and co-immunoprecipitation assays. In addition, we found that the coiled-coil domain of ITSN2 and CC3 region (aa 460–677) of RABEP1 are involved in the interaction. Immunofluorescence analysis shows that ITSN2L and RABEP1 localize in the same subcellular structures. Comparing to co-localized endogenous ITSN2L and RABEP1 (Figure 3, upper panels), excessive GFP-ITSN2Lprotein attracts RABEP1 and the distribution of these proteins shows a completely overlapping pattern (Figure 3, lower panels) similar to other early endosome proteins such as EEA1 [33]. The cellular distribution of these proteins suggests that ITSN2 could be the recruiter of RABEP1 to early endosomes [34].

We also show that overexpression of ITSN2L attenuate the RABEP1 protein level, which could be reversed by proteasome inhibitors. These experiments indicated that ITSN2L promotes RABEP1 degradation and might regulate endocytosis through down-regulating RABEP1 levels. Given the evidence of involvement of ITSN2L in RABEP1 ubiquitination (Figure 4E) [6], we hypothesize that ITSN2L may form a complex with E3 ubiquitin ligase through SH3 domain while interacting with RABEP1 through CC domains respectively. However, which ligase participates in this complex remains to be defined. Therefore, similarly to ITSN1 [35], ITSN2 interacts with RABEP1 and its mutation could result in RABEP1 accumulation and large vesicle formation. Furthermore, RABEP1 can be cleaved by caspase3 [36] during apoptosis and blocks endosome fusion [37]. More recently, RABEP1 was reported regulating cell surface IgE receptor level [38]. Hypoxia-inducible factor (HIF) has also shown to repress RABEP1 transcription, which lead to impaired Rab5-mediated endosome fusion and prolonged EGF receptor half-life [39]. Taken together, the degradation of RABEP1 induced by ITSN2 might have a broad impact on cell physiology such as apoptosis and signal transduction, as well as endocytosis.

Finally, our data demonstrated that ITSN2L and RABEP1 play opposite roles in regulating Tf internalization. ITSN2L overexpression represses Tf uptake while silencing enhances it. On the contrary, RABEP1 elevation promotes Tf endocytosis while knock-down inhibits it. This result confirms previous studies [12,40] Tf uptake is mediated by Tf receptors through clathrin or caveola dependent endocytosis [41]. During those processes, ITSN2L functions as a stabilizing scaffold for the recruitment of endocytic proteins to the site of vesicle formation [8]. High efficiency of endocytosis requires a proper amount of ITSNs [42]. Thus the overexpressed ITSN2L would detain of endocytic proteins and block the formation of endocytic pit [12]. On the other hand, ITSN2 was shown to interact with Cdc42 and activate Cdc42-WASP-Arp2/3 mediated actin polymerization [19]. Our colleagues have also demonstrated that ITSN2 interacts with EPS8 [28], which binds to F-actin and regulates actin dynamics [43]. Hence, ITSN2L knock down might interfere with actin network and result in accumulation of endocytic vesicles [13].

RABEP1 is a key and potent GEF for Rab5 [19,20], which regulates the processes of docking and fusion of endosomal membranes, motility of endosomes and intracellular signal transduction [44]. However, RABEP1 is not functional by itself, it is stably bound to and form a complex with Rabex-5 synergistically activates Rab5 [45]. Thus, overexpression of RABEP1 triggers accumulation of large endocytic vesicles and silencing it will slow down this process. Taking our observations into consideration, a schematic diagram is shown in Figure S3, ITSN2 might regulate endocytosis both directly by itself and indirectly by down-regulation of RABEP1 and function in coordinating vesicle transport and cargo destination determination.

In summary, we show in this study that ITSN2-L interacts with RABEP1, and stimulates its degradation through proteasome pathway. Our data suggest that both of these proteins participate in endocytosis but function in opposing ways. In addition, our results raise the possibility that ITSN2-L regulates endocytosis through altering the protein level of RABEP1.

## 4. Experimental Section

### 4.1. Yeast Two-Hybrid Screens

Vector constructions, siRNAs and antibodies used in this study were described in detail in the Supplementary Materials. Y2H screening was performed using pDBLeu-ITSN2L-CC as bait and pPC86-human brain cDNA library (Invitrogen, Waltham, MA, USA) as prey. Yeast strain MaV203 was sequentially transformed with pDBLeu-ITSN2L-CC and a human brain cDNA library. Approximately 2 × 10^6^ yeast transformants were screened and false-positive clones were excluded by retransforming the prey DNA to MaV203. Positive clones were verified by beta-Gal filter assays and then sequenced.

### 4.2. GST Pull-down Assay

*E. coli* expressed GST and His fusion proteins were purified using Glutathione Sepharose 4B (GE Healthcare, Little Chalfont, UK) or Ni-NTA agarose (Qiagen, Venlo, The Netherlands) according to manufacturer’s instructions. For pull-down assays, 1–5 μg of the GST or GST fusion proteins were mixed with 40 μL of 50% suspension of glutathione-Sepharose 4B beads for 2 h in binding buffer (25 mM HEPES-NaOH (pH 7.5), 12.5 mM MgCl_2_, 10% Glycerol, 5 mM DTT, 0.1% NP-40, 150 mM KCl and 20 mM ZnCl_2_). Then 1–5 mg of His fusion proteins were added for another 2 h incubation. The pellets were washed extensively and resuspended in sample buffer and then analyzed by western blotting using a mouse monoclonal anti-His antibody (GE Healthcare).

### 4.3. Immunoprecipitation and Western Blot Analysis

HeLa cells were maintained according to ATCC’s instruction and transfected in 10 cm dishes using Lipofectamine 2000 (Invitrogen) according to the manufacturer’s instructions with 10 μg of Myc-ITSN2L or Myc-RABEP1. After 24 h, the cells were harvested and lysed in RIPA buffer (50 mM Tris–HCl (pH 7.2), 150 mM NaCl, 1% (*v*/*v*) Triton X-100, 1% (*w*/*v*) sodium deoxycholate, 0.1% (*w*/*v*) SDS and protease inhibitors). The lysates were precipitated using rabbit polyclonal antibodies against Myc-tag (Sigma, St. Louis, MO, USA), and protein A/G plus agarose (Santa Cruz Biotech, Dallas, TX, USA); the immunoprecipitates were separated by 10% SDS–polyacrylamide gels and probed with mouse monoclonal antibodies against RABEP1 (Santa Cruz Biotech), or polyclonal antibodies against ITSN2L, respectively.

### 4.4. Immunofluorescent Analysis

HeLa cells were cultured on glass coverslips in a 12-well plate, grown to 70% confluency and transfected with the indicated plasmids. After 24 h, cells were stained by standard procedures. The primary antibodies used were mouse monoclonal anti-RABEP1 rabbit polyclonal anti-ITSN2L antibodies and anti-Myc antibodies (Santa Cruz Biotech). The secondary antibodies were Alexa-594 or -488 goat anti-mouse antibodies (Molecular Probes, Eugene, OR, USA). The nucleus was stained with Hoechst 33258 (Sigma). The fluorescent signals were analyzed using a fluorescence microscope (Zeiss Axioskop 2, Oberkochen, Germany).

### 4.5. Transferrin (Tf) Internalization Assay

For the Tf uptake assay, transfected cells were washed with serum-free DMEM, and cultured in serum-free DMEM for 1 h, then incubated in DMEM containing 15 μg/mL Alexa Flour 546-conjugated Tf (Molecular Probes) for 15 min. After washing thoroughly with PBS, cells were fixed, permeabilized and incubated with the corresponding anti-Myc, anti-ITSN2L or anti-RABEP1 antibody. Samples were observed under a fluorescent microscope and photos were taken under proper exposure.

## Supplementary Materials

Supplementary materials can be found at http://www.mdpi.com/1422-0067/16/12/26091/s1.
