# Supplementary material for: ITSN2L Interacts with and Negatively Regulates RABEP1
Source: Int J Mol Sci. 2015 Nov 27;16(12):28242–54. doi: 10.3390/ijms161226091 (PMC4691038; doi:10.3390/ijms161226091)
Supplement: Supplementary file 1 [file ijms-16-26091-s001.pdf]

Supplementary Materials: ITSN2L Interacts with and Negatively Regulates RABEP1

Xiaoxu Yang, Feng Yan, Zhicheng He, Shan Liu, Yeqing Cheng, Ke Wei, ShiquanGan, Jing Yuan, Shang Wang, Ye Xiao, Kaiqun Ren, Ning Liu, Xiang Hu, Xiaofeng Ding, Xingwang Hu and Shuanglin Xiang

Vector Construction, siRNA and Antibodies

For Y2H screening, the bait plasmid pDBLeu-ITSN2L-CC was generated by inserting the coding sequence of human ITSN2L-CC in frame with the sequence for the GAL4 DNA-binding domain of pDBLeu (Invitrogen, Waltham, MA, USA). Expression vectors for GFP-ITSN2L, Myc-ITSN2L and HA-ITSN2Lwere constructed by inserting the full-length ITSN2L to pEGFP-N_3 and pCMV-Myc, and the same for RABEP1 to pCMV-Myc (Takara, Kyoto, Japan). Vector pGEX-4T-1 (Amersham, Amersham, UK) was used to construct vectors expressing GST fusion proteins. Thefragments encoding subdomains (Figure 1A) of RABEP1 were cloned in frame into pGEX-4T-1 individually. Plasmid pQE-N_3 (Qiagen, Venlo, The Netherlands) was used to generate vectors expressing His-tagged fusion proteins. The cDNAs encoding subdomains (Figure 1A) of ITSN2L were fused in frame with His tag of pQE-N_3 individually. All constructs have been verified by sequencing. GFP-EEA1 and Myc-EPS8 were cloned by colleagues as reported previously [28]. Three ITSN2L (AF182198.1) siRNAs synthesized by Shanghai GenePharma Co., Ltd. (Shanghai, China) were targeted at 1816–1834 (siRNA A), 1765–1783 (siRNA B), 2033–2051 (siRNA C) from the start codon and the RABEP1 (NM_004703.4) siRNA was targeted at nucleotide positions 1261–1279. ITSN2L antibodies were generated by immunizing rabbits with His-ITSN2L-EHCC derived from human ITSN2L (AF182198.1) (149–692 a.a.). This antibody recognizes human ITSN2 isoforms (both short and long, Figure 2A). The RABEP1 mouse monoclonal antibody was purchased from Santa Cruz (Santa Cruz, CA. USA).

Table S1. ITSN yeast two-hybrid clones. ITSN2L yeast two-hybrid (Y2H) positive clones. Prey, identified ITSN2L-CC binding proteins; GeneID, NCBI gene identification number; Coordinates, positions of the prey proteins that interacted with ITSN2L-CC fragment in Y2H screen; Repeat Numbers, the times of the same gene identified; Potential Domain, possible domain within prey protein that interacts with ITSN2L-CC fragment.

Prey	GeneID	Coordinates (a.a.)	Repeat Numbers	Potential Domain
RABEP1	9135	460–862	7	Coiled coil
EPS15L1	58513	Full-length	7	Coiled coil
Kazrin	23254	Full-length	5	Unknown
SPTBN1	6711	1600–2100	4	Spectrin repeat
NUP62	23636	Full-length	4	Unknown
KCTD10	83892	Full-length	4	BTB
PPFIA1	8500	914–1180	2	Unknown
EPS8	2059	600–810	1	Unknown
PCM1	5108	570–2010	1	Unknown
BICD1	636	576–820	1	Coiled coil

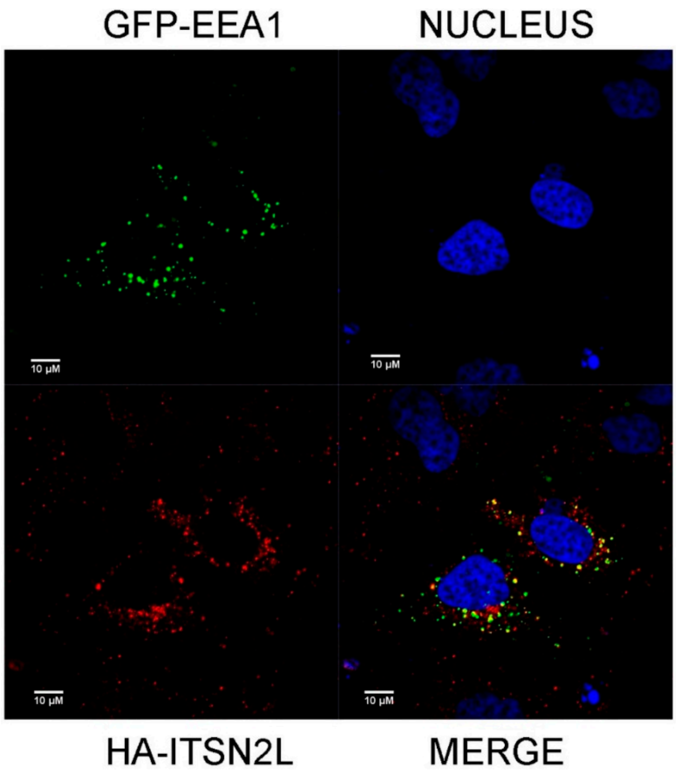

Figure S1. Co-localization of ITSN2L and EEA1. HeLa cells transfected with GFP-EEA1 and HA-ITSN2L were fixed and stained with mouse monoclonal anti-HA antibody.

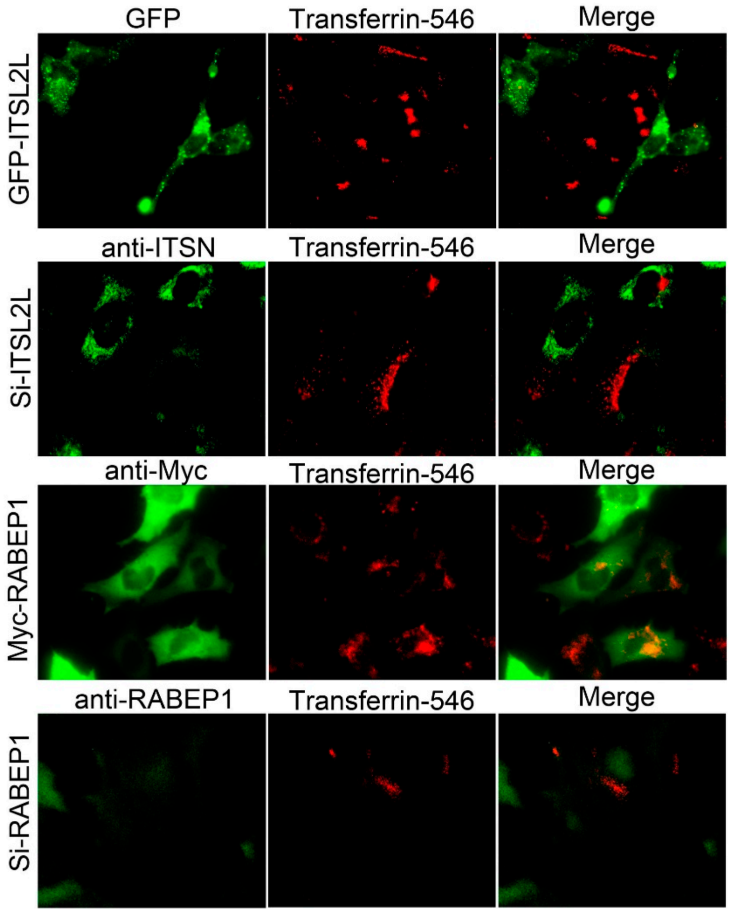

Figure S2. ITSN2L and RABEP1 play opposing roles in Transferrin uptake. High magnification pictures of Figure 6 were shown as indicated.

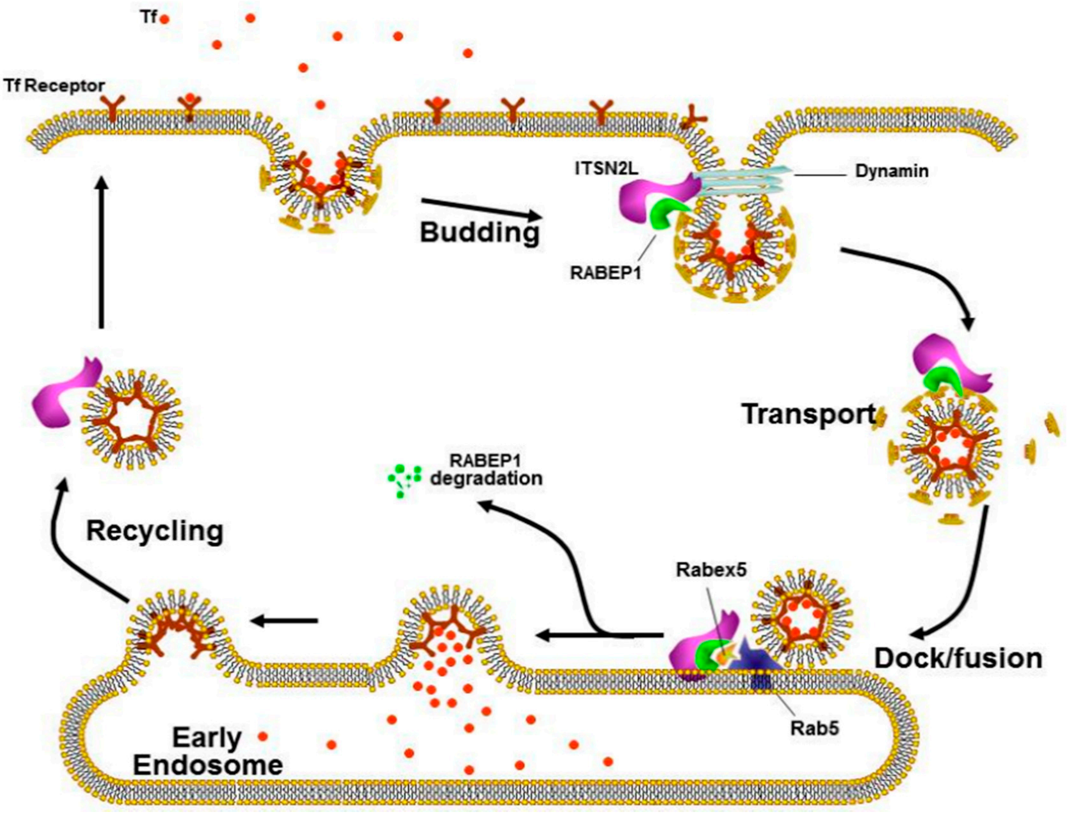

Figure S3. A schematic view of the participation of ITSN2L and RABEP1 in endocytosis.
